# Beyond XAI: a claim-specific translation matrix for cancer biomarkers and therapeutic targets

**DOI:** 10.3389/fonc.2026.1994216

**Published:** 2026-10-29

**Authors:** Vijayachitra Modhukur, Naisarg Patel, Prakash Lingasamy

**Affiliations:** 1 Celvia CC AS, Tartu, Estonia; 2 Department of Obstetrics and Gynecology, Institute of Clinical Medicine, University of Tartu, Tartu, Estonia; 3 Laboratory of Precision and Nanomedicine, Institute of Biomedicine and Translational Medicine, University of Tartu, Tartu, Estonia; 4 Nalam Biosciences OÜ, Tartu, Estonia

**Keywords:** biomarker validation, cancer biomarkers, explainable artificial intelligence, machine learning, multi-omics, precision oncology, SHAP, therapeutic targets

## Abstract

Explainable artificial intelligence (XAI) is increasingly used in molecular and multimodal oncology to identify features that influence machine-learning predictions. A model-important feature may subsequently be described as a biomarker, mechanistic driver or therapeutic target, although these labels represent distinct scientific claims requiring different evidence. We propose the XAI Biomarker Translation Matrix, a claim-specific conceptual framework separating explanation evidence (E), biological evidence (B) and translational evidence (T). A claim gate routes XAI-prioritised candidates into three validation pathways: diagnostic, prognostic or treatment-predictive biomarkers; mechanistic therapeutic targets; and tumour-selective delivery targets. The explanation dimension is XAI-specific and addresses whether attribution is defined, stable, faithful and reproducible, whereas the biological and translational dimensions connect the attributed feature to claim-appropriate evidence. Claim-evidence concordance requires terminology to remain no stronger than the evidence demonstrated for the intended use, and falsification-first translation asks investigators to prespecify the observation most capable of challenging the proposed interpretation. Complementing established AI-reporting, biomarker-validation and target-assessment standards, the matrix adds an XAI-specific explanation-evidence layer and an explicit claim gate linking attribution to claim-specific evidence and terminology: XAI prioritises candidates; the claim determines the validation pathway.

## 1 Introduction

High-dimensional molecular profiling has expanded cancer biomarker discovery from individual genes and proteins to complex genomic, transcriptomic, proteomic, epigenomic and multimodal signatures. Machine-learning approaches can identify predictive patterns and structures in these data, but small sample sizes relative to feature number, correlation among predictors, batch effects and overfitting can make biological interpretation difficult (1, 2). Molecular classifiers can be highly accurate while still relying on signals that are redundant, context-dependent or only indirectly related to disease biology (3). Therefore, predictive performance alone does not establish whether an identified molecular feature represents a biologically meaningful biomarker, a mechanistic dependency, or a clinically actionable target.

Explainable and interpretable machine learning attempts to make such models more transparent. SHapley Additive exPlanations (SHAP), for example, assigns feature-level contributions to model outputs (4), while broader interpretability methods address related questions about model behaviour (5). These approaches can reveal model dependencies, support debugging and reduce a large molecular search space to a tractable set of candidates. However, feature attribution remains a property of a model-data-explanation system rather than direct evidence of molecular causality. Explanation quality therefore requires separate evaluation of stability, faithfulness and sensitivity to the modelling choices that generated the attribution.

The inferential problem begins when attribution is treated as biological validation. A feature may be important to a fitted model without causing the phenotype. A reproducible cancer-associated molecule can be useful as a diagnostic or prognostic biomarker without being required for tumour maintenance. Conversely, a tumour-enriched surface protein can be useful for targeted delivery even if tumour cells are not dependent on its function. These examples illustrate that the same XAI-prioritised feature may support different scientific interpretations and therefore requires different validation strategies depending on the intended claim.

Critiques of *post-hoc* XAI have emphasized that explanations can inherit confounding, instability and artefacts from the model-data system (6, 7). Randomisation and saliency-map evaluations show that apparently plausible explanations may be insensitive to learned model parameters or poorly reproducible (8, 9). Explanation methods can also disagree for the same prediction, while different well-performing models may rely on different feature sets (10–12). These observations motivate an explanation-evidence dimension that asks not only whether an attribution can be generated, but whether it is stable, faithful and robust to reasonable model and explainer alternatives. For intrinsically interpretable models, the same principle applies to the model quantity being interpreted rather than to a *post-hoc* attribution (7).

Correlation is especially important in molecular data. Shapley-value estimates depend on how feature dependence is handled (13), and correlated genomic variables can destabilise or bias feature rankings (14, 15). Biological concordance can strengthen confidence in an attributed signal, as illustrated by overlap between SHAP-prioritised genes and differential-expression signals in an RNA-seq tissue classifier (16), but such concordance does not establish mechanism. Where correlated variables form a coherent module, grouped or pathway-level attribution may be more defensible than interpreting one arbitrarily selected component as biologically dominant.

Recent oncology reviews have already described XAI methods, biomarker discovery and the need for external or biological validation (17). The narrower gap addressed here is the inferential transition after an XAI method nominates a molecular feature: what evidence supports calling that feature a diagnostic or prognostic biomarker, a treatment-predictive biomarker, a mechanistic therapeutic target or a delivery target? Existing standards address neighbouring questions, including AI model reporting and bias, AI-based biomarker readiness, biomarker context of use, clinical actionability and drug-target assessment. The ESMO Basic Requirements for AI-Based Biomarkers in Oncology (EBAI), for example, address AI biomarkers intended for oncology practice (18), while other established frameworks are compared in Section 6. Our aim is not to replace those systems but to make the attribution-to-claim step explicit.

We propose a claim-specific conceptual framework for this intermediate transition. Although several downstream biological and translational principles can generalise to candidates nominated by differential expression, association studies or screening experiments, the present framework is deliberately XAI-centred because model-level attribution is particularly easy to reify as molecule-level biological importance. The framework applies when the explained unit can be linked to a molecular or molecular-composite candidate; purely spatial image heatmaps or tiles without such a biological mapping fall outside its intended scope. The framework remains a conceptual proposal rather than a validated instrument, and its operational criteria require empirical testing.

## 2 From model explanation to a biological hypothesis

XAI primarily answers a model-level question: which inputs contributed to a prediction under a specified model, dataset and explanation procedure? It does not, by itself, establish whether the corresponding molecule is reproducibly altered in independent biological material, whether perturbing it changes a disease-relevant phenotype, or whether measuring or targeting it provides clinical or therapeutic utility.

We therefore separate four questions. First, model contribution asks whether the feature influences model output. Second, biological association asks whether the feature is reproducibly associated with the phenotype in independent biological material. Third, functional relevance asks whether perturbation of the biological entity changes a disease-relevant phenotype. Fourth, translational utility asks whether measuring or targeting the feature is useful in its intended diagnostic, prognostic, treatment-predictive or therapeutic context. We reserve the term treatment-predictive biomarker for evidence that marker status modifies treatment benefit, rather than for a variable that merely contributes to prediction (19, 20). XAI mainly informs the first question; the remaining questions require evidence external to the attribution itself.

## 3 The XAI biomarker translation matrix

We propose describing each XAI-prioritised candidate across three independent dimensions: explanation evidence (E), biological evidence (B), and translational evidence (T). The unit of assessment is a candidate-claim-context combination, because the same molecule can legitimately have different profiles in different tumour types, assay settings or intended uses. E-B-T levels are evidence descriptors, not numerical scores, rankings or regulatory grades. The biological dimension spans association and, where relevant to the claim, functional evidence because both concern properties of the biological entity rather than the model explanation or translational use. A profile such as E3-B1-T0 therefore communicates three different evidence domains rather than three comparable numbers; computational evidence cannot compensate mathematically for absent biological or translational evidence.

The levels described in **Table 1** are conceptual descriptors rather than regulatory thresholds and should be applied with domain-appropriate statistical criteria, assay standards and prespecified decision rules. Within each dimension, levels are interpreted cumulatively: a higher level is assigned only when the lower-level criteria required for that track have also been satisfied. If a higher-order experiment has been performed before a lower criterion is met, that evidence should be reported separately rather than automatically advancing the profile. A minus sign denotes a criterion that was adequately tested but not supported (for example, B1–); ± denotes credible conflicting or mixed evidence; and ? denotes an attempted but inconclusive assessment because of inadequate power, technical failure or other limitations that prevent interpretation. Untested evidence is reported explicitly as not assessed and is distinct from negative, conflicting and inconclusive evidence. Thus, a candidate may retain B2 while recording B3? when a perturbation experiment was attempted but was not interpretable. Dimensional asymmetry reflects qualitatively different evidence domains, so level numbers are interpreted only within their own dimension.

**Table 1:** Operational levels of the XAI biomarker translation matrix.

Dimension	Level	Minimum operational criterion
Explanation (E)	E0	Feature attribution reported without dedicated explanation validation.
	E1	Explanation method, target quantity and relevant baseline/background are explicitly defined.
	E2	Attribution stability and robustness are examined across appropriate resampling, stochastic variation and/or reasonable preprocessing perturbations.
	E3	Faithfulness is evaluated with method-appropriate sanity or ablation tests where applicable; dependence among correlated features and credible model/explainer alternatives are examined, and disagreement is reported.
	E4	A prespecified attribution pattern or candidate set is reproduced in an independent dataset using a comparable explanation pipeline, specifically replicating the attribution rather than only model performance or phenotype association.
Biological (B)	B0	No biological evidence beyond computational attribution.
	B1	The candidate-phenotype association is reproduced in independent biological material with appropriate statistical control and concordant direction.
	B2	The signal is confirmed using an orthogonal molecular measurement, independent assay/platform or, for a composite signature where one-to-one molecular confirmation is not meaningful, fit-for-purpose analytical confirmation appropriate to the composite and claim.
	B3	Track II: an adequately controlled perturbation changes a prespecified disease-relevant phenotype, supporting functional relevance but not by itself a specific mechanism or dependency. Track III: surface/extracellular localisation or accessibility is experimentally confirmed. Track I: this level is not ordinarily required for biomarker validity.
	B4	Track II: convergent perturbation, rescue, pathway-level evidence and/or target engagement supports specificity and a mechanistic/dependency interpretation beyond generic toxicity. Track III: tumour-normal selectivity, target density/heterogeneity and internalisation or shedding are characterised.
	B5	Track II: the functional effect is reproduced in disease-relevant patient-derived, multicellular or *in vivo* models. Track III: target accessibility/selectivity properties are reproduced in disease-relevant patient-derived or *in vivo* models.
Translational (T)	T0	Intended use is undefined or has not been evaluated.
	T1	Context of use and target population are explicitly defined.
	T2	Analytical or target-specific feasibility is demonstrated using fit-for-purpose criteria.
	T3	Independent claim-specific translational validation is demonstrated in the relevant context (for example, external clinical validation for biomarkers or independent target-specific validation for target tracks).
	T4	Track I: high-level clinical validation using a prospective design or a prospectively specified analysis of appropriately archived specimens from a well-designed prospective study/trial, as appropriate to the claim. Tracks II/III: advanced preclinical translational validation, including target engagement, biodistribution and safety as appropriate.
	T5	Track I: independent confirmation at high evidentiary strength (for example, independent/multicentre validation for diagnostic or prognostic claims; for treatment-predictive claims, a prespecified marker-by-treatment interaction in an appropriate comparative trial, with independent confirmation where feasible). Tracks II/III: prospective clinical evaluation or early clinical evidence appropriate to the intended claim.
	T6	Clinical utility, therapeutic benefit or validated targeted-delivery utility is demonstrated in the intended context.

The E, B and T levels above are conceptual descriptors intended to structure inference and reporting; they should not be interpreted as regulatory approval criteria or as equivalent across dimensions.

Levels are cumulative conceptual evidence descriptors; B3-B5 and T4-T5 use claim-specific anchors. Domain-specific thresholds and statistical criteria should be justified for the intended use.

Importantly, a candidate does not need to maximise every dimension. A clinically useful prognostic biomarker need not satisfy the highest mechanistic biological levels, whereas a claim of tumour dependency requires perturbational evidence even if explanation evidence is extensive. Common lower-level criteria can therefore be shared across tracks, while higher-order biological and translational anchors are claim-specific. In particular, mechanistic-target progression depends on perturbation, convergent specificity and disease-relevant functional models, whereas delivery-target progression depends on accessibility, tumour-normal selectivity and target-specific delivery properties rather than oncogenic dependency.

Higher explanation levels (E2-E4) remain important descriptors of confidence in the XAI nomination and are expected whenever attribution is used to select one component over correlated alternatives, to prioritise a candidate specifically because of its explanation profile, or to argue that the model has captured disease biology. A downstream biological or clinical label can therefore be evidence-supported even when explanation stability or external attribution replication remains untested, provided that limitation is reported explicitly. The E dimension therefore chiefly governs confidence in the nomination and the prioritisation of candidates; the claims that rest on the attribution itself, and therefore need higher E levels, are listed in **Table 2**.

**Table 2:** Claim-specific terminology ladders, conceptual minimum E-B-T profiles and worked examples.

Track/example	Profile(s)	Minimum or observed evidence	Claim-appropriate terminology/limit
Track I - diagnostic, prognostic or treatment-predictive biomarker	E1-B0-T0 → E1-B1-T1 → E1-B1-T3 → E1-B2-T5 → E1-B2-T6	Attribution defined → replicated biological association with defined context → independent clinical validation → high-level prospectively designed/specified validation with independent confirmation; treatment-predictive claims require appropriate marker-treatment interaction evidence → demonstrated clinical utility.	Model-important feature → candidate biomarker → independently validated biomarker → clinically validated biomarker → clinically actionable biomarker.
Track II - mechanistic therapeutic target	E1-B0-T0 → E1-B1-T1 → E1-B3-T2 → E1-B4-T2 → E1-B5-T4 → E1-B5-T6	Attribution defined → replicated association/context → adequately controlled perturbation plus target-specific feasibility → convergent specificity/rescue/target-engagement evidence supporting mechanism → disease-relevant models plus advanced preclinical validation → clinical therapeutic benefit.	Model-important feature → candidate target → functionally supported candidate target → mechanistically supported target → validated therapeutic target → clinically validated target.
Track III - tumour-selective delivery target	E1-B0-T0 → E1-B2-T1 → E1-B3-T2 → E1-B5-T4 → E1-B5-T6	Attribution → orthogonal protein confirmation/context → experimentally confirmed accessibility plus target-specific feasibility → disease-relevant selectivity/internalisation with preclinical biodistribution/safety → clinical targeting evidence.	Model-important feature → candidate delivery target → experimentally supported targeting candidate → validated delivery target → clinically validated delivery target.
Attribution-dependent claims (any track)	E2 → E3 → E4, in addition to the B and T profile of the claim’s track	E2: attribution stability and robustness examined. E3: faithfulness tested; correlated features and model/explainer alternatives examined; disagreement reported. E4: attribution pattern reproduced in an independent dataset.	Claims that rest on the attribution itself (selecting one of several correlated features, prioritising a candidate because of its explanation profile, or asserting that the model has captured disease biology) need the corresponding E level; other downstream labels remain at E1.
Illustrative baseline: SHAP-prioritised feature, not examined downstream	E1-B0-T0	Explanation method and attributed feature reported; no independent biological replication or translational evaluation.	Model-important/XAI-prioritised feature. No biomarker or target label is yet supported.
NR5A1 in HGSOC virtual transcriptomics (27)	Track I (prognostic, platinum-response-associated biomarker): E1-B0-T1; B1?; Track II (therapeutic target): E1-B0-T1; B1?	Patient-level cross-validation supports model performance, but no attribution-specific resampling/stability test was reported; therefore E2 is not assigned. B1 is recorded as inconclusive (B1?): the association with platinum response rests on one RT-qPCR cohort of ten stage III HGSOC tumours (four platinum responders, six non-responders) collected independently of the TCGA discovery data, but 18 candidate genes were tested in this small sample without multiple-testing correction (nominal p < 0.05 for NR5A1), so reproduction with appropriate statistical control is not established and B2 is not assigned. All patients in this cohort received platinum-based chemotherapy, so the association is response-associated and no marker-by-treatment interaction was tested. The platinum-treated stage III HGSOC context and the exploratory therapeutic context are defined (T1). Docking/MD remain proof-of-concept and do not establish functional dependency or target engagement.	Track I: model-important/XAI-prioritised feature with preliminary supportive RT-qPCR evidence (B1?); a candidate-biomarker label is not yet supported. Track II: model-important/XAI-prioritised feature; a candidate-target label is not yet supported. The published title’s ‘candidate biomarker and druggable target’ wording would be too strong under this matrix until B1 is established with appropriate statistical control and functional dependency, target engagement and translational validation are demonstrated.
DLL3 in glioblastoma with cyclic-peptide development (28)	Track III (tumour-selective delivery target): E1-B3-T2	Cross-validation and external CGGA validation support performance/generalisation, but no attribution-specific stability analysis across resampling/perturbations was reported; E2 is not assigned. Western blot/confocal microscopy confirm DLL3 expression and surface accessibility, and cell-based peptide-binding/competitive-displacement support target-specific feasibility (B3/T2). *In vivo* biodistribution/safety and disease-relevant delivery validation were not established.	Experimentally supported targeting candidate. Validated delivery target is not yet supported because disease-relevant *in vivo* biodistribution, safety and delivery validation remain pending; explanation stability beyond E1 is also untested.
Venet et al. random gene-expression signatures (22) (non-XAI cautionary comparator)	Not assigned an E-B-T profile	Many random signatures were associated with breast-cancer outcome, illustrating that prognostic association can arise from broad correlated biology rather than a specific causal mechanism.	A prognostic association should not be upgraded to a mechanistic target claim without independent functional evidence.

Lower-level criteria required for each track (Section 3). Within each dimension every level requires all lower levels, except where stated. Track I: B3–B5 are not required for biomarker claims. Track II: B2 is not required, because perturbational evidence (B3) supersedes orthogonal confirmation. Track III: B1 is not required, because orthogonal protein-level confirmation (B2) is the entry criterion. For all tracks E1 is the baseline (see Section 3 for E2–E4). Profiles in the terminology ladders show the minimum level at each terminology step; intermediate levels are implied.

Minimum profiles are proposed reporting anchors rather than validated regulatory thresholds. The lower-level criteria required for each track are listed below the table.

Multi-omic/composite and grouped features. When the XAI unit is a multi-gene signature, cross-modal interaction or biologically grouped set of correlated features, E/B/T assessment applies to the composite as the primary unit. Grouped Shapley or pathway-level attribution can be used when dependence makes component-wise ranking unstable. For a composite, B2 may be satisfied by an independent assay/platform or other fit-for-purpose analytical confirmation of the composite signal when one-to-one molecular measurement is not meaningful; component-level orthogonal validation is required only when a component-specific biological mechanism or target is claimed.

## 4 The claim gate: three validation pathways

The claim gate is the central operational step of the framework (**Figure 1**). Before escalating terminology, investigators specify the intended claim for the candidate in the stated context: diagnostic or prognostic biomarker, treatment-predictive biomarker, mechanistic therapeutic target, or tumour-selective delivery target. The same candidate may therefore enter more than one track, but each claim receives its own evidence profile. This prevents evidence generated for one purpose from being used to imply another.

**Figure 1:**
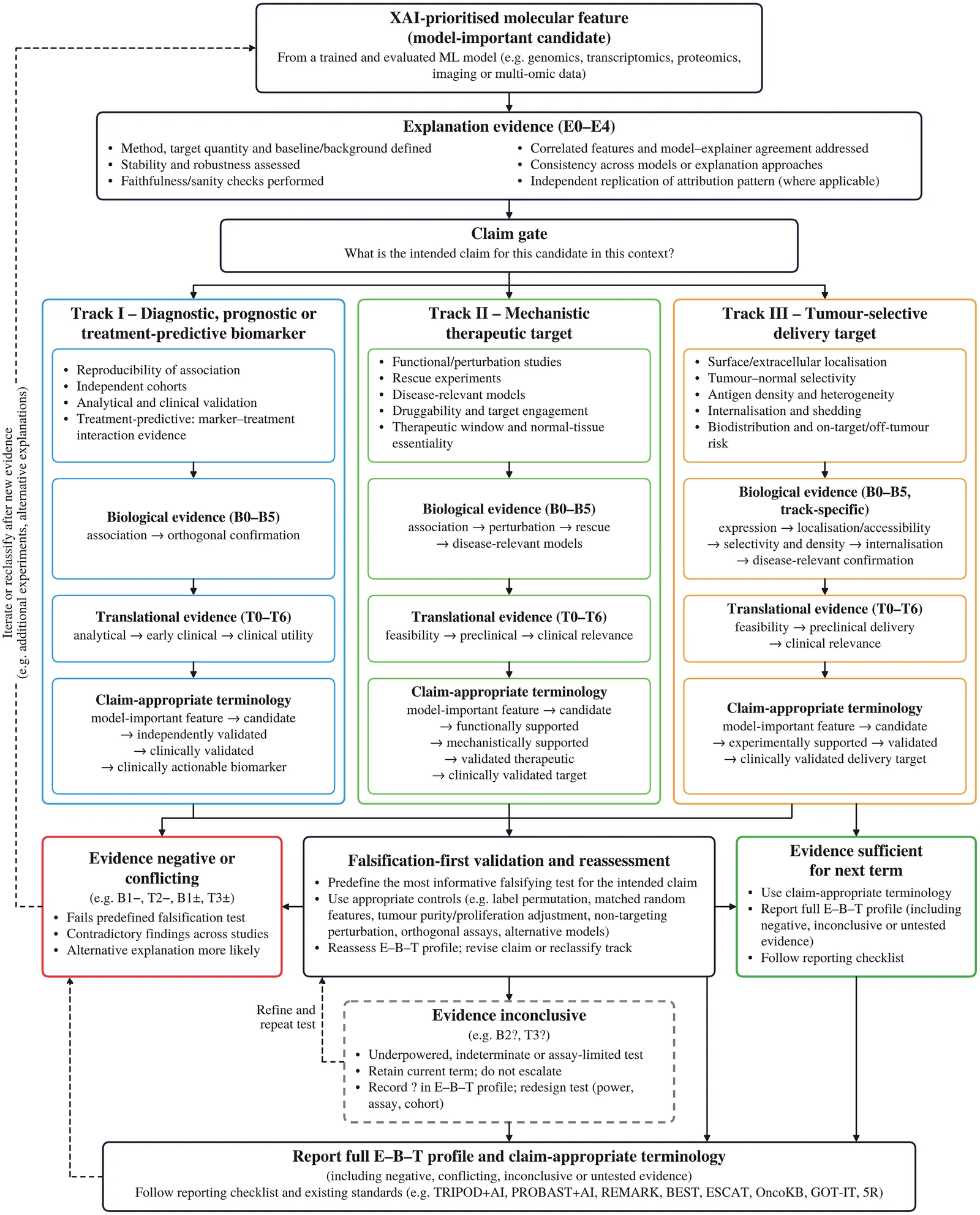
Claim-specific translation after XAI attribution. A claim gate routes an XAI-prioritised molecular feature into diagnostic/prognostic or treatment-predictive biomarker, mechanistic therapeutic-target or tumour-selective delivery-target validation. Explanation (E), biological (B) and translational (T) evidence are assessed throughout the selected track. Claim-evidence concordance constrains terminology, while falsification-first testing and negative or conflicting evidence feed back to the candidate or claim gate without necessarily invalidating model performance. Inconclusive (?) evidence returns to a refined test, without escalating or reclassifying the claim.

Track I - diagnostic, prognostic or treatment-predictive biomarker. A molecular feature may be useful because it reproducibly distinguishes disease states or estimates outcome without being causally involved in tumour biology. The pathway therefore emphasises reproducibility, fit-for-purpose measurement, analytical validity, clinical validity and ultimately clinical utility. A treatment-predictive claim is more specific: it requires evidence that biomarker status modifies treatment benefit, ideally through an appropriate marker-by-treatment interaction analysis rather than outcome prediction alone (19, 20). An explainable model for preoperative relapse prediction in molecularly stratified endometrial cancer illustrates the distinction: SHAP can identify variables contributing to prediction, but individual variables require separate evidence before they are called validated biomarkers or mechanistic drivers (21). Terminology should progress from model-important feature to candidate biomarker, independently validated biomarker, clinically validated biomarker and, only with utility evidence, clinically actionable biomarker.

Track II - mechanistic therapeutic target or dependency. Model contribution does not establish the effect of intervention. A gene may track proliferation, immune infiltration, molecular subtype or tumour purity without itself being required for tumour survival; indeed, broad transcriptomic signatures can be prognostic because they capture dominant biological programmes rather than specific dependencies (22). Track II therefore progresses from a candidate target to a functionally supported candidate target when a verified, adequately controlled perturbation changes a prespecified disease-relevant phenotype. Mechanistic terminology requires stronger specificity, such as convergent perturbations, rescue, pathway evidence or target engagement that argues against off-target effects or nonspecific cellular toxicity. Disease-relevant functional models and translational assessment are then required before escalation to a validated therapeutic target. Target assessment must also consider druggability or tractability, therapeutic window and essentiality in normal tissue, consistent with established target-development frameworks (23, 24). Genome-scale dependency mapping (25) and patient-derived functional models (26) illustrate the qualitatively different evidence produced when candidates are experimentally challenged. The NR5A1 study (27) is a retrospective self-application of the concordance rule: its title used the terms ‘candidate biomarker’ and ‘druggable target’, but under the present framework the evidence supports only a model-important feature with preliminary supportive RT-qPCR evidence, because B1 is inconclusive (18 candidate genes were tested in ten tumours without multiple-testing correction) and no functional-dependency or target-engagement evidence exists. A candidate label would require an adequately powered replication, and a target label direct functional-dependency and target-engagement evidence.

Track III - tumour-selective delivery or targeting molecule. Targeted imaging agents, antibodies, peptides, drug conjugates and radioligands may require an accessible, tumour-enriched molecule even when the tumour is not biologically dependent on its function. Validation should therefore address protein-level localisation, tumour-normal selectivity, antigen or target density, intratumoural heterogeneity, internalisation where required, soluble shedding, normal-tissue expression, biodistribution and on-target/off-tumour risk before escalation to a validated delivery target. The glioblastoma DLL3 study (28) illustrates this distinction: machine learning and SHAP contributed to candidate prioritisation, whereas the evidence supporting a targeting claim arose from downstream structural modelling and experimental binding-related validation rather than from attribution itself.

## 5 Claim-evidence concordance and falsification-first translation

The practical rule arising from the matrix is that terminology should not imply an evidence level beyond that demonstrated for the intended use. We term this claim-evidence concordance. An attribution-only feature is best described as model-important or XAI-prioritised. A replicated molecular association may justify candidate-biomarker terminology; stronger diagnostic, prognostic or treatment-predictive labels require claim-appropriate clinical evidence. For Track II, a single well-controlled perturbation supports functional relevance and the term functionally supported candidate target, whereas mechanistic or dependency terminology requires convergent specificity or rescue-type evidence. A delivery target requires accessibility, selectivity and ligand-relevant evidence but not necessarily oncogenic dependency. Terminology ladders should therefore be interpreted within each track rather than as interchangeable labels. **Supplementary Table S1** provides the claim-specific terminology guide.

The second principle is falsification-first translation. Before validation begins, investigators should prespecify the observation most capable of challenging the intended claim, ideally in a protocol, preregistration or analysis plan when feasible. Appropriate negative controls depend on the track and may include label permutation, matched random feature or gene sets, adjustment for tumour purity or proliferation, alternative model specifications, non-targeting perturbation controls and orthogonal assays. The random-signature phenomenon described by Venet et al. (22) illustrates why a prognostic signal should not automatically be interpreted as a specific mechanism. A criterion should be marked negative only when an adequately powered and technically valid test fails to support it; underpowered, technically unsuccessful or otherwise uninterpretable experiments are recorded as inconclusive rather than refuting the claim. For a biomarker claim, failure of an adequate prespecified association to reproduce should trigger reassessment of cohort effects and explanation robustness; for a target claim, failure of a sufficiently powered perturbation under verified experimental conditions should weaken the functional or dependency claim; and for a delivery target, inadequate selectivity, inaccessible localisation, failed ligand interaction or unacceptable biodistribution should halt escalation.

A negative, conflicting or inconclusive validation result does not necessarily invalidate the predictive model. It may reveal that the model relied on a surrogate, correlated feature or context-specific signal, or simply that the validation experiment was not informative. Evidence flags (−, ±, ?) are defined in Section 3. The highest supported cumulative level is retained while higher criteria are recorded separately (for example, E1-B2-T1; B3?). Such discordance is scientifically informative and should feed back to the candidate or claim gate, prompting reclassification, alternative explanations or additional experiments. A reclassified claim is a new hypothesis that requires its own prespecified falsification test, evaluated in data not used to motivate the reclassification, and the original negative, conflicting or inconclusive result remains part of the reported profile. Closed-loop approaches that return experimental outcomes to AI-guided discovery are increasingly advocated for this reason (29).

## 6 Relationship to existing standards and recent frameworks

The matrix is intended to complement, not compete with, existing standards. TRIPOD+AI addresses transparent reporting of prediction-model studies (30), PROBAST+AI evaluates methodological quality, risk of bias and applicability (31), CLIX-M addresses evaluation of clinical XAI explanations (32), and EBAI defines minimum requirements for AI-based biomarkers intended for oncology practice (18). Established biomarker frameworks additionally distinguish context of use, analytical validity, clinical validity and utility (33–36). Beyond these AI-specific standards, the FDA-NIH BEST Resource formalises biomarker categories and intended uses (37); Simon, Paik and Hayes proposed levels of evidence for prognostic and predictive biomarkers (38); and the Institute of Medicine and NCI criteria established translational expectations for omics-based tests (39, 40).

Oncology actionability and drug-development frameworks address still other parts of the pathway. ESCAT ranks the clinical actionability of molecular alterations (41), while OncoKB and AMP/ASCO/CAP provide structured interpretation of somatic alterations (42, 43); these systems can inform both upper Track II target-actionability levels and treatment-predictive Track I claims involving an alteration-treatment match. GOT-IT and the 5R framework emphasise target validation, assayability, druggability, tissue exposure and safety (23, 24). Recent AI target-identification and closed-loop discovery frameworks address broader computational-to-experimental target-development programmes (29, 44), whereas responsible-AI translational-readiness frameworks focus on whether ML biomarker models are ready for clinical deployment (45). These approaches overlap substantially with parts of the B and T dimensions, which is intentional: the matrix does not claim to replace established biomarker, actionability or target-assessment hierarchies. Its narrower role is to connect an XAI-derived model attribution to the correct downstream evidence system and to prevent evidence for one claim from being used to imply another. **Supplementary Table S2** compares these relationships directly.

The distinctive contribution is therefore not the general statement that XAI requires validation, nor a new substitute for existing clinical-actionability scales. It is the combination of an XAI-specific explanation-evidence dimension, an explicit claim gate, claim-specific terminology control and falsification-driven reassessment. Together these elements define the inferential bridge between ‘the model relies on this feature’ and ‘this feature has earned a particular biological or translational label’.

## 7 Reporting addendum and validation agenda

For molecular-oncology studies that use XAI outputs to make biomarker or target claims, we propose eight reporting elements as a focused addendum to existing guidance. Some refine requirements already implicit in TRIPOD+AI or REMARK, whereas others are specific to the attribution-to-claim transition addressed here:

1. Predefine the intended claim, context of use and unit of assessment (candidate-claim-context). [Refines existing TRIPOD+AI/REMARK reporting of study objectives and intended use.]
2. Specify what the explanation method quantifies, the model output being explained and the relevant reference or background distribution. [Matrix-specific XAI extension.]
3. Assess explanation stability and faithfulness using prespecified, method-appropriate checks. [Matrix-specific XAI extension.]
4. Address correlated predictors, grouped features and credible model/explainer alternatives. [Matrix-specific XAI extension.]
5. Separate replication of the attribution pattern from replication of the biological association and from clinical validation. [Refines external-validation concepts in TRIPOD+AI/REMARK by separating model, biological and clinical replication.]
6. Report orthogonal molecular or target-specific evidence appropriate to the intended claim. [Matrix-specific biological-evidence addition.]
7. Match validation and falsification tests to the claim-specific track, including relevant negative controls. [Matrix-specific claim-matched validation addition.]
8. Report the candidate’s full E-B-T profile, including untested, negative, conflicting or inconclusive criteria, together with an explicit claim-evidence concordance judgement. [Matrix-specific profile and concordance addition.]

A ready-to-use checklist is provided as **Supplementary Table S3** so that authors and reviewers can record the eight reporting elements, the candidate-claim-context, the full E-B-T profile (including untested, negative, conflicting or inconclusive evidence) and the claim-evidence concordance judgement consistently. **Supplementary Table S3** also includes a completed DLL3 worked example showing the supporting study locations, individual criteria, evidence not established and the resulting concordance judgement. The matrix itself should then be empirically tested in a preregistered retrospective audit of molecular-oncology papers using SHAP or related attribution methods to nominate biomarkers, drivers or targets. Two independent raters should assign candidate-claim-context profiles, classify the intended-use track and document negative, conflicting or inconclusive evidence using an operational manual. Inter-rater agreement, ambiguous transitions, external replication, functional perturbation and claim-specific translational validation can then be quantified.

The current framework should therefore be considered testable rather than definitive. It is a proposal for structuring inference, terminology and experimental priorities, not a regulatory score, certification system or substitute for domain-specific assay, biomarker, drug-development or clinical standards.

## 8 Discussion

XAI can make high-dimensional cancer models more scientifically productive by narrowing a large molecular search space into experimentally testable candidates. Its value is greatest when attribution is treated as the beginning of a translational argument rather than its conclusion. The same model-important feature can support different research programmes depending on whether it is being advanced as a diagnostic or prognostic biomarker, a treatment-predictive biomarker, a mechanistic target or a delivery target; those claims require different downstream evidence.

The XAI Biomarker Translation Matrix makes that distinction explicit while deliberately reusing established biological and translational standards where they already exist. Its distinctive contribution is the XAI-specific explanation layer and the routing logic that links attribution to claim-appropriate validation and terminology. Explanation, biological and translational evidence remain independent; claim-evidence concordance prevents terminology from outrunning evidence; and falsification-first testing makes negative, conflicting or inconclusive results part of the profile rather than invisible failures. The framework should be refined empirically through independent rating and worked examples before being treated as an assessment instrument.

The central principle remains concise: XAI prioritises candidates; the claim determines the validation pathway. Applied consistently, this principle could reduce over-interpretation of feature attribution while making XAI-guided oncology studies more experimentally efficient, biologically credible and translationally precise.

## Data Availability

The original contributions presented in the study are included in the article/**Supplementary Material**. Further inquiries can be directed to the corresponding author.
